# Syntactic and non-syntactic sources of interference by music on language processing

**DOI:** 10.1038/s41598-018-36076-x

**Published:** 2018-12-17

**Authors:** Anna Fiveash, Genevieve McArthur, William Forde Thompson

**Affiliations:** 1 Department of Psychology, Macquarie University, Sydney, Australia; 2 Lyon Neuroscience Research Centre, Auditory Cognition and Psychoacoustics Team and Dynamique Du Langage Laboratory, INSERM, U1028, CNRS, UMR5292, Lyon, France; 3 ARC Centre of Excellence in Cognition and its Disorders, Macquarie University, Sydney, Australia; 4 Department of Cognitive Science, Macquarie University, Sydney, Australia

## Abstract

Music and language are complex hierarchical systems in which individual elements are systematically combined to form larger, syntactic structures. Suggestions that music and language share syntactic processing resources have relied on evidence that syntactic *violations* in music interfere with syntactic processing in language. However, syntactic violations may affect auditory processing in non-syntactic ways, accounting for reported interference effects. To investigate the factors contributing to interference effects, we assessed recall of visually presented sentences and word-lists when accompanied by background auditory stimuli differing in syntactic structure and auditory distraction: melodies *without* violations, scrambled melodies, melodies that alternate in timbre, and environmental sounds. In Experiment 1, one-timbre melodies interfered with sentence recall, and increasing both syntactic complexity and distraction by scrambling melodies increased this interference. In contrast, three-timbre melodies reduced interference on sentence recall, presumably because alternating instruments interrupted auditory streaming, reducing pressure on long-distance syntactic structure building. Experiment 2 confirmed that participants were better at discriminating syntactically coherent one-timbre melodies than three-timbre melodies. Together, these results illustrate that syntactic processing and auditory streaming interact to influence sentence recall, providing implications for theories of shared syntactic processing and auditory distraction.

## Introduction

Music and language are two diverse, complex, rule-based systems that on the surface appear extremely different; however, current theory highlights a number of similarities between them. Despite having distinct functions and elementary units, music and language are both characterised by hierarchical structure in which discrete units (words, musical notes) are systematically combined to form larger structural units (sentences, musical phrases). The regularities governing how these elements are combined to form larger hierarchical sequences are collectively known as *syntax*. It has been shown that children quickly learn syntactic rules in their native language^1^ and also develop a detailed understanding of music syntax^2^. In addition, neuroimaging and behavioural research suggest similarities and overlap in syntactic processing between music and language^3,4^, as well as transfer effects between them^5,6^.

There are two prominent theories that suggest music and language share cognitive resources for syntactic processing: the shared syntactic integration resource hypothesis (SSIRH)^4^, and the syntactic equivalence hypothesis (SEH)^3^. The SSIRH suggests that music and language draw upon domain-specific *representational networks* and domain-general *resource networks*. The representational networks are thought to hold information specific to music or language (e.g., verb categories in language, tonal knowledge in music). These networks can be selectively impaired, giving rise to double dissociations that are the hallmark of modularity arguments^7^. In contrast, domain-general resource networks are thought to facilitate the online syntactic integration of elements retrieved from representational networks. Syntactic integration is the process whereby incoming elements are integrated into the currently developing syntactic sequence. The ease of integration is related to how expected the incoming elements are^4^. If an element is unexpected in the syntactic context, more resources are required to integrate it into the sequence. These resource demands are reflected electrophysiologically by an event-related potential (ERP) brain response that occurs approximately 600 milliseconds (ms) after stimulus onset (the P600). The SSIRH is focused on this process of syntactic integration, which is thought to be a relatively “late” syntactic process^8^.

Similar to the SSIRH, the SEH suggests that music and language share syntactic processing resources. It further posits that any syntactically structured sequence (e.g., music, language, action, mathematics) shares resources for syntactic processing that are not shared by semantic processing or by acoustic deviance processing^3^. In addition to integrational processes addressed by the SSIRH (labelled *structural reanalysis and repair* in the SEH), Koelsch (2013)^3^ argues that “early” automatic processes of syntactic *structure building* are also shared between music and language (around 150 ms post stimulus onset). Structure building is considered to be a quick and automatic online process of combining incoming elements (chords, words) into a developing sequence. This early syntactic process depends on initial auditory grouping of elements into a single auditory stream^9,10^. Auditory streaming and early syntactic structure building are considered to be largely automatic, and can occur without attention^9,11,12^. In sum, both the SSIRH and the SEH suggest shared processing between music and language syntax, and predict interference effects (lowered performance) when music and language simultaneously place high demands on syntactic processing resources. These theories have primarily been tested and developed based on experiments introducing syntactic violations or ambiguities into the stimuli, such as out-of-key or unexpected chords or notes in music, and grammatical and semantic errors in language.

Both behavioural and neuroimaging studies have supported the SSIRH and the SEH. For example, behavioural studies have revealed that syntactic violations in sung sentences reduce comprehension for complex sentences^13^, unexpected chords increase reaction times in a lexical decision task^14^, out-of-key chords increase reading times in garden path sentences^15^, and out-of-key chords reduce memory for complex sentences but not word-lists^16^. Interference effects have also been reported from language to music. For example, syntactic garden path sentences presented concurrently with chord sequences reduce judgements of harmonic closure for final notes of a complex chord sequence^17^. Interference in such studies appears to be specific to syntactic processing, as *semantic* anomalies in language^14,15,18,19^ or acoustic deviants in music^13,15,16^ generally do not produce the same interference effect. These findings are therefore compatible with evidence that semantic and syntactic anomalies elicit distinct brain responses (e.g.,^18,20,21^), and that music and language draw on limited-capacity syntax-specific processing resources.

Neuroimaging research has also shown interference effects in the processing of music and language syntax, consistent with both the SSIRH and SEH. Koelsch *et al*.^18^ found that the simultaneous presentation of syntactic violations in music and language produced a lowered left anterior negativity (LAN)—an ERP component elicited with violations in language syntax. In contrast, the N400 elicited by semantic violations in language was not reduced when paired with an out-of-key chord, further suggesting distinct neural processes for syntax and semantics. The authors suggested that the decreased LAN reflected the cost of processing syntax in both domains simultaneously. The same pattern of results was reported by Carrus, Pearce, and Bhattacharya (2013)^19^, where musical expectancy was manipulated (using high and low probability notes) rather than introducing structural violations. In a follow-up to Koelsch *et al*.^18^, Steinbeis and Koelsch (2008)^22^ replicated the finding that the LAN was reduced when concurrently paired with an out-of-key chord, and additionally observed that the early right anterior negativity (ERAN)—an ERP component elicited by errors in music syntax—was reduced in amplitude when paired with violations in language. The authors suggested that the ERAN was reduced in the second experiment and not in the first because in Steinbeis and Koelsch (2008)^22^, participants were asked to pay attention to the music. These studies therefore suggest that the concurrent presentation of demanding music and language syntax creates a processing difficulty that can be observed by a decreased brain response to the violations, and that this effect is enhanced by attention.

Data from fMRI also support the notion that language and music draw upon shared syntactic resources. In one study, participants were presented with complex or simple sung sentences (object-extracted or subject-extracted relative clauses). Sentences were sung on melodies that contained no out-of-key notes, one out-of-key note, or a note with an increase in loudness of 10 decibels^23^. The authors subtracted brain activation to the simple sentences from brain activation to the complex sentences, and found overlap in Broca’s area when the complex sentences were sung with an out-of-key note. This pattern did not occur with the loudness control, suggesting a syntax-specific interaction that occurred with integrational costs. Links between music and language syntax have also been observed at the oscillatory level, where the simultaneous presentation of music and language syntax violations reduced power in the EEG (electroencephalogram) delta-theta frequency band compared to a language-only violation^24^. Therefore, on balance, neuroimaging data also support the view that music and language share limited-capacity processing resources related to syntax.

Based on the literature reviewed above, it appears music and language share resources for syntactic processing that are distinct from acoustic deviance processing or semantic processing. However, other research suggests a more complex interpretation. In particular, some research has shown interference between music syntax and language *semantics*^25–27^. A follow-up to Slevc, Rosenberg, and Patel (2009)^15^ showed that when semantic *garden path* sentences were presented instead of semantic *anomalies*, slower reading times were indeed observed with the simultaneous presentation of out-of-key chords and points of semantic ambiguity^25^. Furthermore, in a lexical decision task, enhanced processing of semantically related compared to unrelated words was reduced when the words were sung on an unexpected chord^26^. These findings suggest that interference effects can also be observed when semantic violations and music syntactic anomalies are combined, making the picture more complex.

To further investigate whether interference effects were specifically related to shared syntactic processing resources, Van de Cavey, Severens, and Hartsuiker (2017)^28^ contrasted syntactic garden path sentences with syntactic anomalies while participants were concurrently listening to pitch sequences that contained “clusters” of related notes. Cluster shifts (moving from one group of related notes to another group of related notes) were introduced either concurrently or non-concurrently with the unexpected sentence element. The authors found that the phrase (or cluster) boundaries of the music were processed more shallowly when participants were reading the garden path sentences than when they were reading sentences with a syntactic error, suggesting reduced resources available for processing the phrase boundary in music. This study suggests that the shared processing resources between music and language are related to processes of *reintegration*, rather than specifically related to rules of syntactic combination. This view is also discussed by Slevc and Okada (2014)^29^, and can explain the findings of interference with semantic garden path sentences in Perruchet and Poulin-Charronnat (2013)^25^. This study also raises the question of whether a syntactic *violation* or *ambiguity* is necessary to engage processes of syntactic integration and to observe interference between music and language. Because the evidence supporting both the SSIRH and SEH is based on violation paradigms, it is so far unclear whether concurrent syntactic processing of music and language without violations or ambiguities draws on the same processing resources, or whether this interaction is only observed when syntactic violations or ambiguities are introduced.

Violation paradigms are also problematic because interference could be explained by the recruitment of additional shared error detection mechanisms rather than shared syntactic processing resources per se. Further, it is unlikely that strong violations of syntax will occur in natural listening conditions, and so such paradigms offer little to theories of background music and language processing. Although manipulations of melodic expectancy can be used to avoid categorical violations of musical structure (e.g., the work by Carrus *et al*.^19^ based on computational models of musical probability^30^ and the work by Kunert *et al*.^23^), such manipulations still introduce violations of expectation which could trigger error detection mechanisms. Further, they involve difficulties in structural integration that are also unlikely to occur in natural listening conditions. To control for the possibility that syntactic violations are merely distracting, previous studies have included a control condition that does not involve a syntactic violation but matches the level of distraction of the syntactic violation. Such conditions have involved a change in timbre^15,16^ or loudness^13,23^. If interference is observed for syntactic violations but not for changes in timbre or loudness, then interference is interpreted as syntax-specific.

However, it has been noted that out-of-key notes or chords are usually more distracting than control stimuli because they typically violate both sensory *and* tonal expectations, whereas control stimuli only violate sensory expectations^31^. Although it is possible to manipulate tonal expectancies while controlling for sensory factors (e.g., by manipulating melodic expectation), it is still challenging to match levels of distraction between control and experimental stimuli. Further, timbre and loudness are powerful cues to auditory streaming^9^, and so changes in these attributes may be processed by the brain as a new stream of information, disrupting the coherence of the musical stimuli. In contrast, out-of-key notes can be close in pitch to other melody notes and integrated into the perceptual stream, even when they violate syntactic expectations. Thus, studies to date have failed to convincingly match the level of distraction associated with an out-of-key note or chord. This problem raises the possibility that syntactic violations in music and language lead to interference effects not because of shared capacity-limited syntactic processing resources, but because syntactic violations are highly distracting and engage error-detection mechanisms. This alternative interpretation underscores the need to evaluate shared syntactic resource models without introducing stimuli with syntactic violations, and to explore the effects of syntax in conjunction with processes of (non-syntactic) auditory distraction.

To investigate these gaps in the literature, and to place syntactic processing within a larger cognitive framework, we evaluated predictions from the SSIRH and the SEH combined with auditory distraction theory from the *changing-state hypothesis*. The changing-state hypothesis predicts that auditory distraction arises from the obligatory serial processing of changes in background auditory stimuli that interfere with processing of the concurrent task, rendering changing-state stimuli more distracting than steady-state stimuli^32–34^. This hypothesis suggests a distinction between local violations in a sequence (suggested to capture attention momentarily and which can be controlled with top-down attention) and changing-state stimuli, which interfere directly with the processing of sequences in memory^32,34^. The changing-state hypothesis has primarily been used to explain interference by background speech on serial recall; however, it has been shown that tones interfere with serial recall in a similar way^35^, and that the violation of expectations of changing-state stimuli also contributes to auditory distraction^36^. Drawing from the syntactic processing and auditory distraction frameworks, we manipulated distraction and syntactic structure across four auditory conditions. We focused on background melodies and environmental sounds *without* semantic content (lyrics or words), to focus specifically on the effect of syntactic interference and auditory distraction on word reading and recall. Experiment 1 was designed to investigate the effect of different background auditory conditions on recall for sentences and word-lists. Based on results of Experiment 1, Experiment 2 was designed to investigate the relationship between syntactic processing and auditory streaming. The combination of these experiments allows us to elucidate different factors that contribute to interference effects when music and language are combined.

## Experiment 1

Experiment 1 explored the effects of background auditory stimuli on syntactic and non-syntactic language processing. Four types of auditory stimuli were presented concurrently with visually presented sentences or word-lists, and the extent to which these stimuli interfered with language recall was assessed. We reasoned that greater errors in recall should indicate that the auditory stimuli must be taxing cognitive resources needed for reading, either through auditory distraction, syntactic interference, or a combination of these processes. The following auditory conditions were created to manipulate levels of syntactic interference and auditory distraction. Sequences were considered syntactic if they contained a structured melody that followed melodic rules based on music theory (e.g., melodies that contained clear progressions, small movements between adjacent notes, and allowed for predictions of upcoming notes). Sequences were considered distracting if they involved (a) unpredictable changes in timbre, or (b) unpredictable changes in pitch and timing that meant the participant could not easily predict the upcoming note or timbre. Manipulations of distraction were based on research from the changing-state hypothesis suggesting that continual changes that violate expectations have a distracting effect on serial recall because they capture attention and interfere with sequencing processes^34,36^. Unpredictable changes not only capture attention but may place a burden on syntactic processes that attempt to determine structure from complex input.

The first type of auditory stimuli consisted of *one-timbre* melodies with syntactic structures that conformed to expectations, were played with a piano timbre, and were considered minimally distracting (predictable syntax, low distraction). The second condition used *scrambled* melodies that disrupted global syntactic structure and hence did not conform to expectations (unpredictable syntax, high distraction). The disruption of global syntactic structure through the unpredictability of pitch and timing should logically make these stimuli more distracting than the one-timbre melodies, and they may also place a high burden on syntactic processes that attempt to represent structure from unfamiliar input. The third condition was a *three-timbre* melody condition (predictable syntax, high distraction). In this condition, each note in the one-timbre melodies was randomly played on one of three different instruments. Based on the changing-state hypothesis, persistent changes in timbre were expected to increase the level of distraction. The fourth condition, *environmental sounds*, consisted of background soundscapes (such as ocean or jungle soundscapes) that had no syntax or unpredictably changing elements, and hence should theoretically be less distracting than other conditions (no syntax, low distraction).

We hypothesised that *sentence recall* should be susceptible to both syntactic interference and auditory distraction effects, but *word-list recall* should only be susceptible to the effects of auditory distraction, and not syntactic interference, given that word-lists have no syntactic structure. Both music and sentence processing require online structure building to create a syntactic representation of the sequence^4^. If these resources are shared between music and language, the syntax in the music should interfere with the online structure building in the sentences, therefore resulting in a poorer syntactic representation and reduced recall. We included word-lists to provide a baseline to assess the effect of background auditory stimuli on the recall of a sequence of non-syntactic words.

We had two main reasons for measuring recall as a dependent variable. First, we wanted a sensitive measure of syntactic and non-syntactic language processing. Other measures, such as comprehension accuracy or grammatical error detection, are only suitable for sentences, and often focus attention to a specific point in time rather than across the whole sequence (e.g., local error detection). Second, traditional measures such as sentence comprehension implicate several linguistic processes, and it is difficult to disentangle the precise elements being measured. Memory for sentences and word-lists allow us a sensitive measure of one aspect of language processing that reflects reading success. Sentence recall has also proven to be a sensitive measure of syntactic processing (e.g.^16,37^) and has previously been used to investigate syntactic representations in children with specific language impairment^38–40^. To recall a sentence accurately, participants must parse the sentence, analyse the thematic relations (i.e., the order of events), and interpret the underlying syntactic structure^37^. Considering the length of the sentences (10–16 words), it would have been essential for participants to syntactically process the sentence in order to recall all of the words. For the word-lists in which syntactic relationships were absent, it would have been necessary for participants to use serial recall alone. Recall was therefore a sensitive dependent variable to assess contributions of syntactic interference and distraction across sentences and word-lists. To enhance ecological validity, sentences and word-lists were presented in full on the screen, as in natural reading conditions. Previous work has used word-by word^15,17,25^ or syllable-by-syllable^14^ presentation which does not reflect natural reading conditions. Therefore, memory for sentences and word-lists should reflect the success of reading while processing background auditory stimuli. We predicted that auditory conditions with *distracting* elements should interfere with recall of both accompanying sentences and accompanying word-lists. However, auditory conditions with *syntactic* structure should only interfere with recall of accompanying sentences, because sentences also have syntactic structure. We also predicted that the effects of distraction and syntax should be additive.

In relation to our specific conditions, we predicted that the one-timbre melodies, scrambled melodies, and three-timbre melodies would interfere with sentence recall more than the environmental sound condition, but that the same pattern would not be observed for word-list recall. Predictions for the scrambled melody condition were ambiguous, given the relative lack of research using stimuli in which individual tones were scrambled. We considered the possibility that scrambling melodies might disrupt global structure and hence result in an incoherent stimulus that does not engage syntactic resources. If so, then scrambled melodies should be a source of distraction (affecting word-list recall), but should not lead to syntactic interference (affecting sentence recall). However, the adjacent tones in our scrambled melodies were within the pitch range expected for syntactic melodies, and could be grouped by auditory streaming mechanisms. Therefore, it is also possible that scrambled melodies might encourage the cognitive system to vigorously engage syntactic resources in order to discover underlying structure. If so, then the scrambled condition might yield both a distraction effect and syntactic interference on sentence recall, as well as a distraction effect on word-list recall.

Our three-timbre condition was based on the changing-state hypothesis^32^, therefore our initial prediction was that continual changes in timbre should be more distracting than a *steady-state* timbre. However, because timbre changes have a powerful influence on auditory streaming (e.g.^9^), it is also possible that alternating timbres will disrupt the coherence of the incoming auditory stream, and disengage the process of syntactic structure building^41^. If the degree of syntactic processing is similar for the one-timbre and three-timbre melody conditions, then we should only observe effects of distraction. However, if the timbre changes inhibit syntactic integration, then it is also possible that three-timbre melodies will interfere less with sentence processing than one-timbre melodies. As there is limited research on how timbre changes in a melody affect syntactic processing, our initial predictions were based on the *changing-state* hypothesis. The current auditory stimuli therefore allow us to observe the differential effects of syntactic structure and auditory distraction, as well as potential links with auditory streaming, on reading and recall of sentences and word-lists.

### Results

As can be seen in Table 1, the majority of errors recorded for both sentences and word-lists across all auditory conditions were errors of omission. The sum of errors, referred to as the composite score, was calculated for each condition and analysed. Given the low error rates for non-omission errors, analyses of individual error types were not conducted.

**Table 1:** Breakdown of Recall Error Types.

Sentences	One-Timbre		Scrambled		Three-Timbres		Environmental	
	*M*	*SD*	*M*	*SD*	*M*	*SD*	*M*	*SD*
Omissions	1.41	1.03	1.64	0.10	1.13	0.95	0.71	0.53
Substitutions	0.21	0.15	0.24	0.13	0.18	0.13	0.19	0.13
Variants	0.08	0.09	0.09	0.10	0.06	0.06	0.05	0.06
Additions	0.33	0.24	0.37	0.22	0.24	0.20	0.16	0.20
Reversals	0.11	0.16	0.15	0.16	0.15	0.20	0.07	0.10
**Word-lists**								
Omissions	0.38	0.30	0.49	0.34	0.38	0.32	0.35	0.28
Substitutions	0.06	0.05	0.05	0.06	0.05	0.07	0.04	0.05
Variants	0.14	0.11	0.15	0.13	0.11	0.09	0.10	0.09
Additions	0.13	0.12	0.15	0.13	0.14	0.13	0.12	0.10
Reversals	0.12	0.12	0.19	0.19	0.11	0.12	0.10	0.12

Note: For all auditory and sentence types, the largest errors in recall were “omission” errors. Values refer to the average number of errors per word-list/sentence across all trials.

A repeated measures (RM) analysis of variance (ANOVA) showed a main effect of language type, *F*(1, 49) = 102.75, *p* < 0.001, η^2^ = 0.68, a main effect of auditory condition, *F*(3, 147) = 55.66, *p* < 0.001, η^2^ = 0.52, and an interaction between language type and auditory condition, *F*(3, 147) = 23.69, *p* < 0.001, η^2^ = 0.33. Considering the main effect of language type (as expected from the difference in word length), we conducted separate paired-sample analyses for sentences and word-lists.

#### Sentences

**Figure 1:**
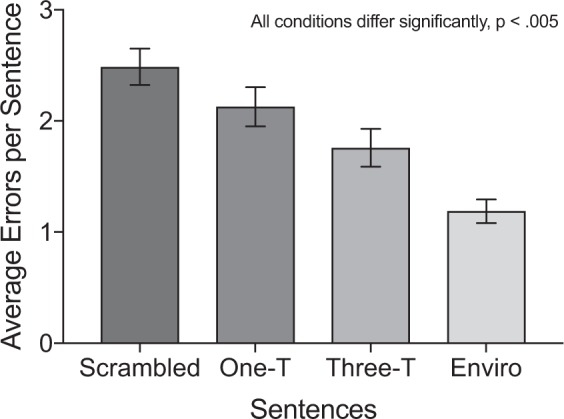
Average error scores for sentence recall across the four auditory conditions. One-T refers to the one-timbre condition, three-T refers to the three-timbre condition, and enviro refers to the environmental sound condition. An average error score of one indicates that participants made, on average, one error per sentence. Error bars indicate one standard error either side of the mean.

Error scores for sentence recall across the four auditory conditions are presented in Fig. 1. There were significant differences between each condition, as confirmed by pairwise comparisons with Holm-Bonferroni corrected *p* values (*p’*) reported for six comparisons. The scrambled melody condition (unpredictable syntax, high distraction; *M* = 2.49, *SD* = 1.16) was associated with more errors in sentence recall than the one-timbre melody condition (predictable syntax, low distraction; *M* = 2.13, *SD* = 1.25), *t*(49) = 3.57, *p’* = 0.002, *d* = 0.51, the three-timbre melody condition (predictable syntax, high distraction; *M* = 1.76, *SD* = 1.20), *t*(49) = 7.00, *p’* < 0.001, *d* = 1.00 and the environmental sound condition (no syntax, low distraction; *M* = 1.19, *SD* = 0.75), *t*(49) = 10.20, *p’* < 0.001, *d* = 1.58.

Looking beyond the scrambled condition, an interesting pattern was observed. As predicted, the environmental sound condition was associated with significantly fewer errors than both the one-timbre melody condition, *t*(49) = 6.83, *p’* < 0.001, *d* = 1.09, and the three-timbre melody condition, *t*(49) = 5.12, *p’* < 0.001, *d* = 0.86. However, the one-timbre melody condition was associated with significantly more errors than the three-timbre melody condition, *t*(49) = 3.70, *p’* = 0.002, *d* = 0.52. This finding contrasts with the changing-state hypothesis, which predicts that the primary consequence of alternating timbres should be distraction. Instead, it appears that the alternating timbres disrupted syntactic processing of melodies, resulting in reduced syntactic interference with sentences. This result will be discussed further in the Discussion below.

#### Word-lists

**Figure 2:**
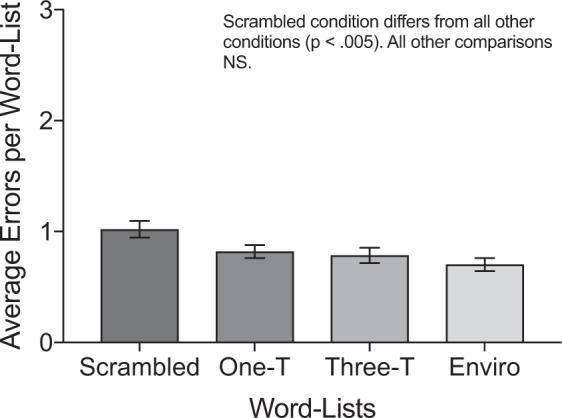
Average error scores for word-list recall across the four auditory conditions. One-T refers to the one-timbre condition, three-T refers to the three-timbre condition, and enviro refers to the environmental sound condition. An average error score of one indicates that participants made, on average, one error per word-list. Error bars indicate one standard error either side of the mean. NS = non-significant.

Error scores for word-list recall in the four auditory conditions are presented in Fig. 2. Pairwise comparisons (Holm-Bonferroni corrected *p* values reported for six comparisons) revealed that the scrambled melody condition (*M* = 1.02, *SD* = 0.53) resulted in significantly more errors than the other three conditions: one-timbre melody (*M* = 0.82, *SD* = 0.42), *t*(49) = 3.63, *p’* = 0.004, *d* = 0.52, three-timbre melody (*M* = 0.79, *SD* = 0.49), *t*(49) = 3.99, *p’* < 0.001, *d* = 0.55, and environmental sounds (*M* = 0.70, *SD* = 0.41), *t*(49) = 5.51, *p’* < 0.001, *d* = 0.81. The other differences between conditions were not significant: one-timbre and three-timbre melodies, *t*(49) = 0.61, *p’* = 0.55, one-timbre melodies and environmental sounds, *t*(49) = 2.34, *p’* = 0.07, three-timbre melodies and environmental sounds, *t*(49) = 1.42, *p’* = 0.32, suggesting that recall was similar across these three conditions for the word-lists. These findings differ from those observed for sentences, and confirm that syntactic interference was observed for sentence recall but not word-list recall. Nonetheless, error rates were considerably lower for word-lists than for sentences, raising the possibility that the pattern of results observed for word-lists partially reflects a ceiling effect. This possibility is considered in the Discussion section.

#### The scrambled condition

We were interested to find that the scrambled condition resulted in the poorest recall for both sentences and word-lists. This result could be interpreted in two ways. First, the unpredictable changes in the randomised notes may have been too distracting to perform the language task and hence led to poorer performance compared to the other conditions. This interpretation would explain why the scrambled condition resulted in significantly poorer performance for both sentence and word-list recall. To isolate effects of auditory distraction and syntactic interference, we used word-list error scores as a baseline (auditory distraction effects only), and calculated the difference score of sentence minus word-list errors for each participant. By subtracting errors in word-list recall from errors in sentence recall, the difference score should reflect a measure of syntactic interference as separate from distraction effects. A repeated measures ANOVA conducted using the difference scores indicated that the main effect of auditory condition was significant, *F*(3, 147) = 23.69, *p* < 0.001, η^2^ = 0.33. Pairwise comparisons (Holm-Bonferroni corrected *p* values reported for six comparisons) showed no significant difference between the one-timbre (*M* = 1.31, *SD* = 1.11) and scrambled melody (*M* = 1.47, *SD* = 0.96) conditions, *t*(49) = 1.31, *p’* = 0.20, suggesting that the main difference between these two conditions may be in the higher level of distraction for the scrambled melody stimuli. There was also a significant difference between the one-timbre and three-timbre melody (*M* = 0.97, *SD* = 0.91) conditions, *t*(49) = 2.92, *p’* = 0.01, *d* = 0.43, suggesting that the lower error scores in sentence recall for the three-timbre melody condition compared to the one-timbre melody condition was an effect of syntax, not merely an effect of auditory distraction. All other comparisons remained significantly different: one-timbre melody and environmental sounds (*M* = 0.49, *SD* = 0.64), *t*(49) = 5.39, *p’* < 0.001, *d* = 0.81, scrambled and three-timbre melodies, *t*(49) = 4.65, *p’* < 0.001, *d* = 0.66, scrambled melody and environmental sounds, *t*(49) = 7.13, *p’* < 0.001, *d* = 1.04, and three-timbre melody and environmental sounds, *t*(49) = 4.11, *p’* < 0.001, *d* = 0.60.

A second explanation for why the scrambled condition resulted in the poorest recall is that participants may have tried to impose a syntactic structure on the sequence, therefore engaging syntactic processing resources to a greater extent. We did not intend for the scrambled condition to contain syntactic *violations*; however, it is possible that by disrupting global syntax, we also violated temporal and melodic expectations. By definition, violations of expectancies vary with the complexity of melodies. To confirm that the scrambled sequences were more complex than one-timbre sequences, we used the MIDI toolbox^42^ to calculate melodic complexity for the one-timbre and scrambled melodies, based on pitch and rhythmic aspects of the MIDI files^43^. According to this model, and confirmed by a paired-samples *t*-test, the scrambled melodies (*M*_complexity_ = 4.19, *SD* = 1.88) were significantly more complex than the one-timbre melodies (*M*_complexity_ = 3.83, *SD* = 0.22), *t*(29) = 30.12, *p* < 0.001, *d* = 1.74. This difference in complexity suggests that another reason scrambled melodies resulted in the poorest sentence recall is because of increased use of syntactic processing resources to try and make sense of the unexpected notes. Considering the difference scores and melodic complexity ratings together, it is likely that the scrambled condition both increased distraction *and* increased syntactic processing effects.

### Discussion

Experiment 1 investigated the effects of four different auditory conditions (one-timbre, scrambled, three-timbre, and environmental) on recall for complex sentences (complex syntax) and word-lists (no syntax). As predicted, participants made significantly more errors in sentence recall when the sentences were paired with one-timbre melodies (predictable syntax, low distraction) or three-timbre melodies (predictable syntax, high distraction) than when they were paired with environmental sounds (no syntax, low distraction). These effects were not evident for the word-lists. These outcomes suggest that syntactic interference can occur between music and language processing even without syntactic violations.

The results of Experiment 1 provided us with two further insights. First, the scrambled condition (unpredictable syntax, high distraction) resulted in the poorest recall across both sentences and word-lists. An analysis on the difference between sentence and word-list error scores suggested that the scrambled condition was more distracting than the other conditions. A complexity analysis revealed that the scrambled melody condition was also more melodically complex than the one-timbre melody condition. This difference in complexity suggests that the scrambled condition may have engaged syntactic resources to a greater extent as participants tried to make sense of the more complex and unpredictable incoming information. We suggest a combination of these two factors that should be teased apart in future research.

Second, Experiment 1 revealed that although the three-timbre melody condition was associated with significantly more errors than the environmental condition (as expected), it also led to significantly *fewer* errors than the one-timbre condition. As this effect did not occur for word-lists, and persisted when the baseline error scores for word-lists were subtracted from errors in sentence recall, it appears that the alternating timbres changed the way participants processed the syntax in the music, and reduced the interference effect when paired with language. This result might have occurred because the changing timbres disrupted auditory streaming processes^9,41^, resulting in a less coherent syntactic representation of the melody. This disrupted representation may have freed up resources for syntactic processing of sentences. It is also possible that the alternating timbres resulted in attention being drawn to the timbre changes in the music, rather than the syntax of the music, thereby reducing interference from music syntax processing on sentences.

These possibilities led to the prediction that alternating timbres within a melody results in poorer syntactic processing of that melody. The only remaining interpretation is that the three-timbre melodies were less distracting than the one-timbre melodies. Although this possibility is counterintuitive, the low error rates in word-list recall make it difficult to rule out this unlikely interpretation. Experiment 2 was designed to evaluate the role of syntactic processing and distraction effects for sequences with a single timbre or alternating timbres. To this end, participants were presented with pairs of melodies that consisted of one-timbre or three (alternating) timbres. If alternating between three different timbres in a melody inhibits syntactic processing of that melody, then same-different judgements for three-timbre melodies should be worse than same-different judgements for one-timbre melodies. However, if three-timbre melodies are less distracting than one-timbre melodies, then same-different judgements should be enhanced for the three-timbre melodies than for the one-timbre melodies. Experiment 2 tested these hypotheses.

## Experiment 2

It has been hypothesised that stimuli that are perceptually grouped as originating from different sources, or stimuli that change state (e.g., change acoustically in some way) require more attention to process (changing-state hypothesis)^33^. It has also been suggested, in both the changing-state hypothesis, and with Gestalt grouping principles, that incoming auditory information is grouped via auditory streaming processes into meaningful units, and that this process has a cognitive processing cost^9,44,45^. When combined, these hypotheses suggest that the three-timbre condition in Experiment 1 may have both induced an increased cognitive cost, and led to an impaired syntactic representation of the melody, because of disrupted auditory streaming processes^9,41^. Given that a syntactic sequence can only emerge from a coherent auditory stream of musical elements, any disruption of auditory streaming should limit the level of syntactic processing that occurs. The weakened music syntax processing, in turn, should free up resources for processing *language* syntax, resulting in improved sentence recall.

To test this suggestion, a same-different paradigm was used in Experiment 2 to investigate whether music syntax is processed to a greater extent in one-timbre melodies compared to three-timbre melodies. If listeners are more sensitive to syntactic changes in one-timbre melodies compared to three-timbre melodies, this result would suggest that changing timbres interrupt and reduce syntactic processing. This result would explain why the three-timbre condition was associated with fewer errors in sentence recall than the one-timbre condition in Experiment 1. Further, if syntactic processing is reduced in the three-timbre condition, this would suggest an important interplay between auditory streaming and syntactic processing at an early stage of processing. More generally, investigating the relation between auditory streaming and syntactic processing may contribute to an understanding of the extent to which music and language draw upon shared neural resources.

### Results

#### Sensitivity analyses

A paired-samples *t*-test revealed that the d’ values for the one-timbre melody condition (*M* = 0.74, *SD* = 0.46) were significantly higher than the d’ values for the three-timbre melody condition (*M* = 0.55, *SD* = 0.43), *t*(40) = 3.08, *p* = 0.004, *d* = 0.48. This finding suggests that participants were more sensitive to differences between melodies in the one-timbre melody condition compared to the three-timbre melody condition.

A RM ANOVA on only the *same* trial accuracies depending on the pairing manipulation showed no main effect of timbre, *F*(1, 40) = 0.06, *p* = 0.94, a main effect of pairing type, *F*(1.68, 67.17) = 20.75, *p* < 0.001, η^2^ = 0.34 (Greenhouse-Geisser correction reported as the assumption of sphericity was violated χ^2^(2) = 8.27, *p* = 0.02), and no interaction effect, *F*(2, 80) = 1.87, *p* = 0.16.

For the *different* trial accuracies, there was a main effect of timbre, *F*(1, 40) = 13.18, *p* = 0.001, η^2^ = 0.25, a main effect of pairing type, *F*(2, 80) = 73.57, *p* < 0.001, η^2^ = 0.65, and no interaction effect, *F*(2, 80) = 1.15, *p* = 0.32 (See Supplementary Material, Figs 1 and 2). Pairwise comparisons are reported in Supplementary Material, Table 1. These results suggest that (a) the three-timbre condition reduced detection of different trials in particular, (b) differences between melodies were detected more accurately when one melody included a violation (as expected), and (c) same trials were detected more poorly when both melodies included a violation.

#### Reaction times

A paired-samples *t*-test revealed that the mean RT (ms) for the one-timbre melody condition (*M* = 694.7; *SD* = 266.81) was significantly shorter than the three-timbre melody condition (*M* = 754.7; *SD* = 265.20), *t*(40) = 3.86, *p* < 0.001, *d* = 0.60.

### Discussion

Experiment 2 showed that differences in syntax between two melodies were detected more quickly and accurately when played with a single instrument compared to alternating instruments. Melodies with alternating timbres were included in Experiment 1 to explore the effects of combined syntax and distraction in musical stimuli, in line with the changing-state hypothesis^32,33^. However, the results of Experiments 1 and 2 combined suggest that alternating timbres may reduce syntactic processing in music, possibly through both a disruption to the auditory stream, and less attention directed to syntactic processing. This reduced syntactic processing may free up resources to process language (as observed in Experiment 1), and make it more difficult to compare syntactic structure between two melodies (as observed in Experiment 2). It should be noted that decreased performance on the same-different task in the three-timbre condition would also be predicted by the changing-state hypothesis, as alternating timbres should increase the level of distraction of the stimuli. However, when combined with the findings from Experiment 1, the current pattern of results suggests a disruptive effect of timbre on auditory streaming processes, resulting in decreased syntactic processing. Further research investigating one-timbre and three-timbre melodies using ERPs has revealed that the ERAN response to an out-of-key note in a melody is significantly reduced in three-timbre melodies compared to one-timbre melodies^46^. This result, combined with the current findings, strongly suggests that disrupting an auditory stream with alternating timbres reduces syntactic processing in the brain. These findings therefore highlight the important connection between auditory streaming and syntactic structure building at early stages of processing, which can help inform more general auditory processing models.

## General Discussion

The results of the current experiments suggest that syntactic interference effects can be elicited without violations of syntactic structure, and that auditory distraction and auditory streaming also influence the concurrent processing of music and language. These findings help to clarify theories of shared syntactic processing between music and language, and suggest that the concurrent processing of music and language reveals interference at a higher level than shared error detection mechanisms.

In Experiment 1, we found that memory for complex sentences (but not word-lists) was significantly decreased when participants were concurrently listening to one-timbre melodies compared to environmental sounds. Interestingly, melodies with alternating timbres resulted in *better* sentence recall than one-timbre melodies, though participants still performed better in the environmental sound condition. This finding was at first surprising, as the changing-state hypothesis predicts that melodies with alternating timbres should be more distracting than melodies that do not alternate in timbre. Our interpretation is that alternating timbres disrupted processes of syntactic structure building by continuously interrupting auditory streaming, thereby reducing the capacity of music to interfere with language syntax processing.

Experiment 2 provided indirect support for this hypothesis, showing that melodic same-different judgements were more accurate when melodies were played with one instrument compared to three instruments. Presumably, melodies played with one instrument are subject to greater syntactic processing and hence, deeper encoding. However, the result also aligns with the changing-state hypothesis (e.g., stimuli that change state take more resources to process)^33,44,45^, in that performance is likely to be worse for highly distracting stimuli than for non-distracting stimuli. Nonetheless, the combined results of Experiments 1 and 2, and recent results from Fiveash *et al*. (2018)^46^ favour the notion that interrupting auditory stream formation with alternating timbres results in impaired syntactic structure building, leading to a weaker syntactic representation^9,10^. In other words, Experiments 1 and 2 suggest that syntactic interference effects are distinct from basic distraction effects. If they were purely distraction effects, we should have observed an additive effect of alternating timbres and syntactic interference. Therefore, the current pattern of results does not support the changing-state hypothesis in its entirety, but rather supports the interpretation that changing-state stimuli and syntactic structure interact in the processing of auditory stimuli.

The finding that sentence recall was better in the three-timbre melody condition than the one-timbre melody condition in Experiment 1 suggests that alternating timbres disrupted a source of interference by music on sentence processing. This result has implications for the SEH, as it suggests that early processes of auditory streaming influence the shared structure building processes in music and language. Future research could continue to investigate this connection. It would also be interesting to investigate whether interruptions to the auditory stream only interfere with initial structure building stages, or whether interruptions to the auditory stream also influence later processes of syntactic integration, such as discussed in the SSIRH. It has been shown that disrupting auditory streams with alternating timbres disrupts early syntactic processing^46^; however, future research could investigate this effect at later stages as well.

The current results begin to uncover important connections between auditory streaming and syntactic processing. According to models of music perception (e.g.^10^), feature extraction and grouping (auditory streaming) is a necessary stage before syntactic structure building. Therefore, a disruption to auditory streaming through alternating timbres should result in reduced syntactic processing. The current results therefore support previous research suggesting that syntax appears to be a level of processing that is reliant on low-level auditory scene analysis and segregation^10^. Previous research investigating tone sequences that differ in timbre between notes has also shown that alternating timbres can result in auditory streaming that renders the sequence non-cohesive^41^; however, to our knowledge, this is the first experiment to show such an effect in melodies, and to show a reduced syntactic interference effect when combined with sentence stimuli.

An interesting finding from Experiment 1 was that the scrambled condition led to the poorest performance in both sentence and word-list recall. There are two possible reasons for these results. First, it is possible that the scrambled condition primarily resulted in auditory distraction effects, owing to its unpredictable temporal and melodic nature^36^. The finding that scrambled melodies resulted in significantly poorer recall than the other auditory conditions for word-lists supports this interpretation. In addition, difference scores for sentence minus word-list recall showed no difference between the scrambled and one-timbre melody conditions, suggesting a distraction as opposed to syntactic effect. Second, it is also possible that the non-conforming and unpredictable nature of the scrambled condition resulted in increased syntactic processing. Complexity measures (taking into account pitch and duration information) showed that the scrambled condition was more “complex” than the one-timbre condition^43^. These possibilities are not mutually exclusive, and it is possible that the scrambled condition led to the poorest performance in recall across both sentences and word-lists precisely because the manipulation of syntax engaged both syntactic processing resources and resulted in general auditory distraction effects. Future research should try to tease apart the distinction between distraction, complexity, and syntactic complexity, to ensure studies that increase syntactic complexity are in fact measuring syntactic interference, and not just greater engagement of general processing resources. To further investigate the effects of auditory stimuli on sentence and word-list processing, future research could systematically manipulate background auditory stimuli by holding all other parameters constant, but changing, for example, the level of distraction based on pre-defined parameters. It would also be interesting to evaluate baseline performance with no background stimuli.

Methodologically, it was difficult to directly compare sentence recall with word-list recall because sentences had more words, and therefore a greater margin for error. It is therefore possible that factors other than the difference in syntactic structure may have led to the observed differences between sentences and word-lists, such as potential floor effects for errors in word-list recall. Considering that there were five words in each word-list, average error scores between 0.7–1.02 errors per word-list in Experiment 1 do not appear to reflect a floor effect; however, future research could explore different word-list lengths to increase error rate. Regardless of this limitation, errors in sentence recall and the subtraction of errors in word-list recall from errors in sentence recall provide a clear picture of the effects of the different auditory conditions on language processing. One way to make sentence and word-list recall more comparable in future research is to match the number of content words in the sentences and the word-lists, and only score recall for content words. This procedure was adopted in Baddeley, Hitch, and Allen (2009)^47^ but was not suitable for the current study for two reasons. First, in our sentence stimuli, the object-extracted and subject-extracted sentence structures required more content words than the sentences used in Baddeley *et al*.^47^. If we had matched the content words in the sentences and word-lists, word-lists would have been too long to recall, especially with added background auditory stimuli. Second, we scored recall for both function and content words, because function words are important to syntactic relationships between elements. Nonetheless, future research could explore alternative scoring methods for comparing word-list and sentence recall.

The current results have implications for the literature on reading with accompanying background music. A recent review outlined the conflicting evidence for the effect of background music on reading, and concluded that current theories of auditory distraction cannot account for the pattern of results observed in the literature^48^. The review compares predictions from a number of different hypotheses that predict when auditory stimuli should influence reading, including the changing-state hypothesis, the phonological-interference hypothesis, the semantic-interference hypothesis, and the interference-by-process hypothesis. However, it does not include models of shared syntactic processing which could account for interference effects of structured background music on sentence reading. The current results suggest that sentence recall (as a measure of reading success) is influenced by both syntactic structure and changing-state stimuli, but importantly, that these two elements interact. This finding suggests that the relationship may be more complex than previously understood. In particular, the current research suggests that contributions of both concurrent syntactic processing and processes of auditory streaming should not be overlooked in future theories of auditory distraction and reading.

## Conclusion

The results of this study provide important evidence concerning the nature of syntactic processing resources that are shared between music and language, and how auditory streaming plays a crucial role in syntactic processing. Establishing interference effects *without* violations of syntax is a crucial experimental finding, as the results cannot be explained merely by shared error-processing mechanisms. Instead, syntactic interference effects in this context can be attributed to music and language drawing on a shared pool of limited-capacity syntactic processing resources, providing support for theories of shared syntactic processing, including the SSIRH and the SEH. Our findings further suggest that syntactic processing is dependent on successful auditory streaming, and have implications for future theories of auditory distraction. These results fit within a larger framework proposing domain-general syntactic processing resources in the brain.

## Methods

All studies were conducted in accordance with the Declaration of Helsinki, and were approved by the Macquarie University Human Research Ethics Committee (5201500300). All participants gave written informed consent to participate.

### Experiment 1

#### Participants

Fifty-four native-English speakers from Macquarie University participated in this study for course credit. Four participants were excluded: two due to recording error, and two due to error rates more than three standard deviations above the mean. This left 50 participants (*M*_age_ = 22.5, range: 18–68, 42 females). Participants had an average of 4.39 years of private music lessons (range: 0–28 years). All participants reported listening to music, with an average listening time of 112 minutes per day (*SD* = 55.72 mins). Sample size was calculated using a G*Power analysis^49^ that considers effect sizes obtained in published research involving comparable conditions. Fiveash and Pammer (2014)^16^ found a small effect of background stimulus type on recall across both word-lists and sentences in a repeated measures ANOVA (η^2^ = 0.05). Based on this effect size, and an α of 0.05, a sample size of 43 participants was required to achieve power of 0.95 (calculated using G*Power)^49^. We therefore aimed to recruit approximately 50 participants for our sample. This sample size is within the range used in previous syntactic interference studies (e.g., 32 in^14^; 96 in^15^). All participants were tested before data were scored, eliminating any chance of “optional stopping”^50,51^.

#### Design

The experiment was a 2 (language: sentences, word-lists) by 4 (auditory condition: one-timbre, scrambled, three-timbre, environmental) within-subjects design.

#### Auditory stimuli

**Figure 3:**
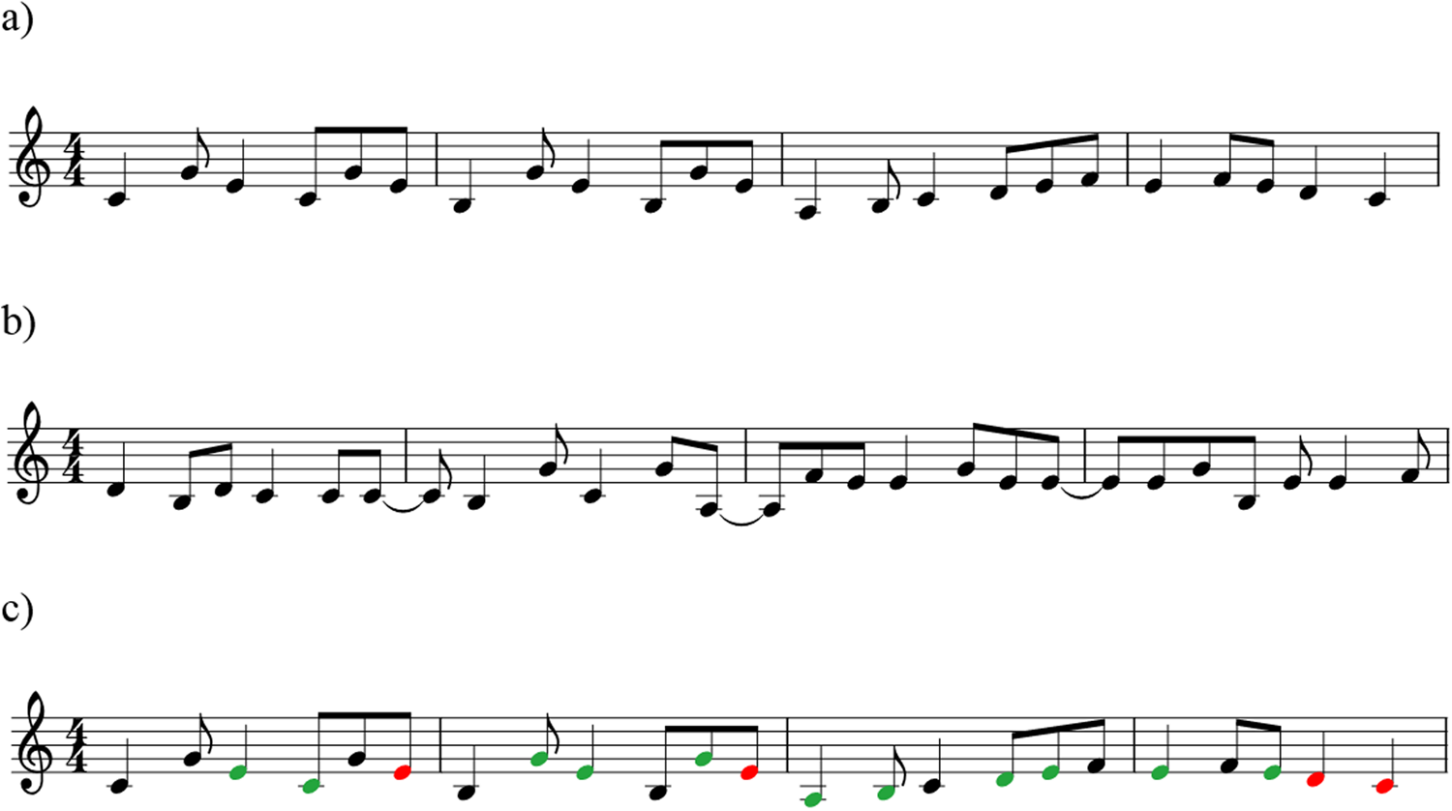
(**a**) one-timbre melody, (**b**) scrambled melody, (**c**) three-timbre melody (where black = piano, green = guitar, red = vibraphone). Not pictured: environmental sounds.

The *one-timbre melody* condition (predictable syntax, low distraction) consisted of 30 single line musical instrument digital interface (MIDI) melodies that were unfamiliar and composed by a professional composer (the last author). All stimuli were 8–9 seconds long, 120 beats per minute (bpm), were composed in the keys of C, G, D, and A major, and had an average of 23.9 notes (range: 18–30 notes). Melodies were played through MIDI instrument Steinway grand piano and exported using GarageBand. See Fig. 3a.

The *scrambled melody condition* (unpredictable syntax, high distraction) was created by taking each note and its duration from each one-timbre melody and randomising note order. Each melody in the one-timbre melody condition therefore had a scrambled version with the same notes, note durations, and overall duration, but with disrupted global syntax (both melodic and rhythmic syntax were disrupted). Scrambled music has previously been used as a “non-syntactic” comparison condition, as it holds constant the total acoustic information available, but disrupts syntactic structure^52,53^. However, in previous work, scrambled stimuli were created by splicing recordings into 250–350 ms chunks of sound, regardless of whether the segmentation interrupted individual notes or chords, and then re-splicing these segments in a random order. Thus, the procedure not only scrambled syntactic structure, but also disrupted the processing of individual tones and chords every 250–350 ms, undermining fundamental mechanisms of auditory streaming. As such, observed differences in responses to fully syntactic stimuli and re-spliced scrambled stimuli could reflect differences in syntactic processing, differences in the engagement of auditory streaming, or both. In contrast, we employed randomised stimuli that disrupted syntactic structure, but retained all of the discrete elements contained within the original melodies (see Fig. 3b).

The *three-timbre melody condition* (predictable syntax, high distraction) was included to maintain syntactic structure and to increase distraction. To achieve this, the notes in the one-timbre melodies were played through three different MIDI instruments (Steinway grand piano, acoustic guitar, and vibraphone). An external random number generator was used to determine which of the three instruments would play each note, making the three-timbre sequences unpredictable in relation to timbre. No instrument played more than three notes in a row. See Fig. 3c.

The *environmental sound condition* (no syntax, low distraction) was included as a control condition. Environmental stimuli were downloaded from the www.sounddogs.com website. Interested readers are directed to this website for specific examples of stimuli. Thirty background environmental sounds (approximately 9 seconds long to match melody duration) were chosen as background ambient or common sounds (e.g., jungle background, train station ambience, ocean noises etc.). Stimuli were normalised for loudness in respect to the other stimuli, and were selected to contain no salient distracting features.

#### Language stimuli

There were two types of language stimuli: complex sentences and word-lists. Complex sentences contained an object-extracted relative clause, such as: *The scout who the coach punched had a fight with the manager*. This construction is more difficult to parse than: *The scout who punched the coach*, because it contains a long-distance dependency between *who* and *punched*^54^. Sixty object-extracted sentences (10–16 words, 50–68 characters) were adapted from^55^ and^13^. Interested readers are directed to these papers for specific examples of stimuli, and full stimuli can be provided on request. Sixty, five-word word-lists (30–33 characters, 8–10 syllables) were created from randomisations of the sentences’ content words (e.g., scout, director, helped, fought, assisted). Content words from the sentences were randomised across word-lists such that the word-lists were composed of words from many different sentences. It was further ensured that there were no semantic links between words in the word-lists. Word-lists were created this way to try and maintain a similar level of difficulty across the sentences and word-lists. Five-word word-lists were chosen based on research showing that short-term memory is limited in its capacity for recall of unrelated elements^56^, and previous research that used five-word word lists in a similar design^16^. As there were not quite enough content words to finish the 60 word-lists, (as some content words in the sentences were repeated) extra words were taken from the word-lists in^16^.

#### Procedure

Participants first read and signed the information and consent form, and then completed a brief musical education and preference questionnaire. Participants were told they would hear four different types of auditory stimuli that would be paired with either a sentence or a word-list, and that their task was to recall the sentence or word-list out loud, in the correct order, once it disappeared from the screen. Participants were given practice trials where they heard examples of the four auditory conditions, and read and recalled sentences and word-lists. After the practice trials, the experimenter confirmed verbally and through participant responses that the participant understood the task, and the experiment proper began.

**Figure 4:**
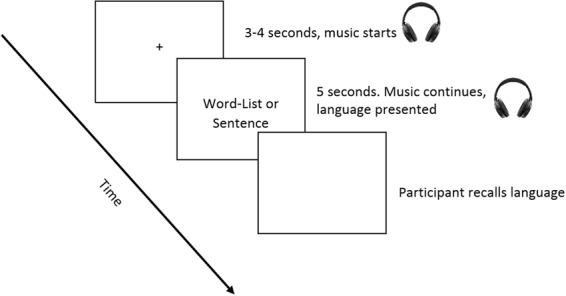
Timing of stimulus presentation. Headphones represent auditory stimulus presentation.

For each trial, the auditory stimulus started playing first, and then the language stimulus was presented on the screen for five seconds. The auditory stimulus started first so that participants had time to develop a representation of the auditory stimulus before the language appeared. The word-list or sentence was presented on the screen horizontally in one line, and word-lists were separated by commas. Auditory and language stimulus presentation ended simultaneously. Once the stimuli disappeared, the word *Recall* appeared on the screen, and participants recalled out loud what they could remember before pressing spacebar for the next trial. Auditory and language stimuli were randomised for each participant so that the 30 stimuli in each auditory condition were randomly paired with 15 word-lists and 15 sentences. The timing of stimulus presentation for each trial is shown in Fig. 4. There were 120 trials in total. Stimuli were presented via Matlab^57^ (version 2014b) using Psychtoolbox^58^ (version 3.0.12).

#### Scoring

Sentence and word-list recall was scored in a similar way to previous research (e.g.^39,59^), where the sum of all omissions, substitutions, variants, additions, and reversals was calculated for each trial (see Table 2). For sentences, both content and function words were included in the scoring, as we were interested in recall of the full syntactic sequence. For word-lists, reversals were included as errors because participants were explicitly instructed to recall the words in the correct order. The average error score in each condition for sentences and word-lists was then calculated. Each type of error was evenly weighted in the error score calculation, receiving a score of one. Higher scores reflect more errors, and therefore lower recall of the language stimuli. All stimuli were blind scored so that markers were unaware of condition. One marker scored the responses from all participants and a second marker scored the responses from 31 of 50 participants. The markers agreed on 94.4% of responses. Any discrepancies were discussed and a mutual agreement was reached.

**Table 2:** Scoring of Sentences and Word-lists.

Error Type	Description
Omissions	No recall.
Substitutions	Word substituted for another. E.g., synonyms, or close in pronunciation.
Variants	Same word, just different variants of it. Also used if one letter was different.
Additions	Words not in original stimuli.
Reversals	Word in wrong position. If following words were in the same *relative* order after the reversal, these were scored as correct.

#### Analysis

To determine whether the auditory conditions had a different effect on sentence recall compared to word-list recall, we first ran a repeated measures analysis of variance (ANOVA) with the factors language type (sentences, word-lists) and auditory condition (one-timbre, scrambled, three-timbres, environmental sounds). Given that sentences comprised more words than word-lists, it was possible that sentences would be associated with higher error scores (omissions and inaccurate recall) than word-lists because of the scoring procedure. We therefore expected a main effect of language type; however, we predicted that we would also find a main effect of auditory condition and an interaction between language type and auditory condition. We expected both syntactic interference and distraction effects to be observed in recall of sentences, and only distraction effects to be observed in recall for word-lists. Because of the difference in stimulus length, any main effects or interaction effects were explored separately in sentences and word-lists using Holm-Bonferroni corrected pairwise comparisons (adjusted *p* values represented by *p*’). Cohen’s *d* effect sizes for these comparisons are reported based on repeated measures data, taking into account correlations between conditions.

### Experiment 2

#### Participants

Forty-three participants were recruited from Macquarie University and participated for course credit. Two participants’ data were lost due to technical errors, leaving 41 participants (*M*_age_ = 22.10 years, age range: 18–70, 30 females). Participants had an average of 4.5 years of private music lessons (range: 0–20 years). All participants reported listening to music, with an average listening time of 144 minutes per day (*SD* = 107.7 mins). Previous same-different experiments included a range of sample sizes (e.g., sample sizes of 30, 14, 21, and 64 respectively)^60–63^, and there are no existing studies that compare melodies with alternating timbres in one auditory stream as in the current study. We therefore ran a G*Power analysis with a medium-to-large effect size, (d_z_ = 0.65), and an α of 0.05 which determined 33 participants were needed to achieve power of 0.95. Given our uncertainty about the size of the effect, we tested an additional 10 participants to ensure we had adequate power to detect an effect. Data collection was finalised before data were analysed, ensuring there was no optional stopping^50,51^.

#### Design

Experiment 2 was a same-different task, with a 2 (melodies: same, different) by 2 (music condition: one-timbre melody, three-timbre melody) within-subjects design.

#### Stimuli

The melodies were the same as Experiment 1, which were played either with a piano (one-timbre melody condition) or with three alternating instruments (three-timbre melody condition). In half the trials, the melodies were the same, and in half the trials they were different. In *different* trials, two melodies could differ by an *altered* note (i.e., a nearby in-key note) or by a *violation* note (i.e., a nearby out-of-key note). Note changes were always on the first or third beat (the strong beats), and in the second or third bars of the four bar melodies. The altered and violation manipulations were included to ensure optimal sensitivity to syntactic processing, as detecting an altered note that is in-key has been shown to be more difficult than detecting an altered note that is out-of-key^64^. Including both manipulations therefore provided a range of difficulty in same-different judgements.

**Figure 5:**
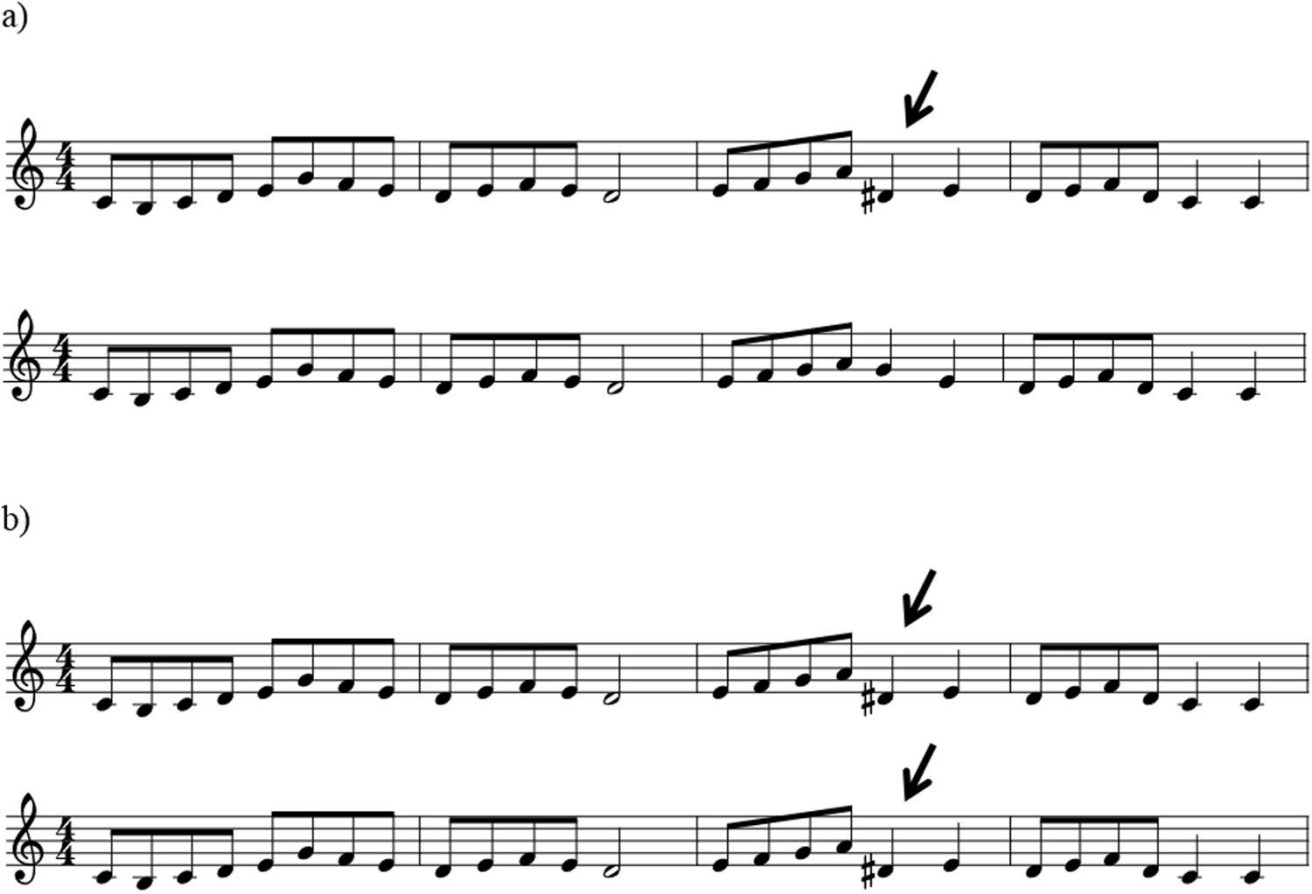
Examples of melodies in the same-different paradigm. (**a**) is an example of a violation-original *different* trial. (**b**) is an example of a violation-violation *same* trial.

The pairings were created using Audacity software, and there was a 2-second break between melodies, as in the Montreal Battery of Evaluation of Amusia (MBEA)^65^. Same-different pairs had six different possible combinations: original-original (same), altered-altered (same), violation-violation (same), original-altered (different), original-violation (different), altered-violation (different). For each of the pairings, melodies were presented in both possible orders (e.g., original-violation and violation-original). See Fig. 5 for an example.

#### Procedure

Participants first read and signed the information and consent form, and then completed a brief musical education and preference questionnaire. Participants were instructed that they would hear two consecutive melodies that would either both be played by piano alone, or by alternating timbres. They were told that the timbres would not change, that there would only be a one-note difference (either in-key or out-of-key) between melodies, and that they should indicate whether there was a difference by pressing the same (z) or different (m) key on the keyboard. Practice trials contained examples of both the three-timbre and one-timbre conditions, as well as examples of altered and violation melodies. After ensuring that the participant understood the task, the experiment proper began. The experiment consisted of 60 one-timbre melody pairs and 60 three-timbre melody pairs (i.e., 120 trials in total) presented via Matlab^57^ and Psychtoolbox^58^. All pairings were randomised so that order of presentation was different for each participant. Participants had a break after every 30 trials. The whole process took approximately 50 minutes.

#### Analysis

To calculate how sensitive participants were to differences in stimuli for the one-timbre and three-timbre melody conditions, d prime (d’) values were calculated using signal detection theory^66^. D prime measures sensitivity to signal versus noise without response bias. A *hit* was recorded when the correct response was different, and the participant answered different. A false alarm was recorded when the correct response was same, and the participant answered different. Z scores were calculated, and z (false alarms) were subtracted from z (hits) to calculate the d’ value for each participant in the one-timbre and three-timbre melody conditions. Average reaction times (RTs) were also calculated, and trials that were more than 3 *SD* above the grand RT mean were excluded from average RT score calculations. To assess whether there were differences in sensitivity (d’ scores) and RT (ms) in same-different judgements between the one-timbre and three-timbre conditions, we ran paired-samples *t*-tests.

To investigate whether there was a difference across the different melody pairings (e.g., original-original, original-violation), we calculated raw accuracy scores for each of the six pairings. Two RM ANOVAs were then run for (a) the same trials and (b) the different trials, with the within-subject factors of timbre (one-timbre, three-timbres) and pairing type (same: original-original, altered-altered, and violation-violation; different: original-altered, original-violation, altered-violation). This analysis was conducted to test the assumption that melodies including violations would be more easily differentiated, and to investigate whether the different pairings produced different results for the one-timbre compared to the three-timbre condition. Reaction times were not calculated because of the small number of trials in each condition (*n* = 10).

## Electronic supplementary material

Supplementary Information

## Data Availability

All stimuli are available from the corresponding author for academic use. Group data can be made available upon request; however, individual data is protected under the ethics agreement of the Macquarie University Research Ethics Committee.
