# Supplementary material for: Syntactic and non-syntactic sources of interference by music on language processing
Source: Sci Rep. 2018 Dec 17;8:17918. doi: 10.1038/s41598-018-36076-x (PMC6297162; doi:10.1038/s41598-018-36076-x)
Supplement: Supplementary file 1 — Supplementary Information [file 41598_2018_36076_MOESM1_ESM.docx]

**Supplementary Material**

Syntactic and non-syntactic sources of interference by music on language processing

Anna Fiveash^1,2,3*^, Genevieve McArthur^3,4^, and William Forde Thompson^1,3^

1. Department of Psychology, Macquarie University.
2. Lyon Neuroscience Research Centre, Auditory Cognition and Psychoacoustics Team and Dynamique Du Langage Laboratory, INSERM, U1028, CNRS, UMR5292.
3. ARC Centre of Excellence in Cognition and its Disorders, Macquarie University.
4. Department of Cognitive Science, Macquarie University.

*Corresponding author:

Email: anna.fiveash@inserm.fr

*Supplementary Figure 1.* Accuracy ratings for the *same* trials used in Experiment 2 for both one-timbre and three-timbre conditions: original-original, altered-altered, violation-violation.

*Supplementary Figure 2.* Accuracy ratings for the *different* trials used in Experiment 2 for both one-timbre and three-timbre conditions: original-altered, original-violation, altered-violation.

| Supplementary Table 1 | | | |
| --- | --- | --- | --- |
| *Pairwise Comparisons for Different Melody Pairs, Experiment 2* | | | |
| Pair | Mean Difference | *t* | *p* |
| Same |  |  |  |
| 1T-orig-orig  1T-alt-alt | -0.02 | -0.70 | .49 |
| 1T-orig-orig  1T-viol-viol | 0.13 | 3.09 | .004* |
| 1T-alt-alt  1T-viol-viol | 0.15 | 5.19 | < .001** |
| 3T-orig-orig  3T-alt-alt | 0.06 | 2.02 | .05 |
| 3T-orig-orig  3T-viol-viol | 0.20 | 4.66 | <.001** |
| 3T-alt-alt  3T-viol-viol | 0.14 | 3.26 | .002* |
| 1T-orig-orig  3T-orig-orig | -0.05 | -1.53 | .13 |
| 1T-alt-alt  3T-alt-alt | 0.03 | 0.89 | .38 |
| 1T-viol-viol  3T-viol-viol | 0.02 | 0.56 | .58 |
| Different |  |  |  |
| 1T-orig-alt  1T-orig-viol | -0.23 | -5.88 | <.001** |
| 1T-orig-alt  1T-alt-viol | -0.31 | -12.93 | <.001** |
| 1T-orig-viol  1T-alt-viol | -0.09 | -3.21 | .003* |
| 3T-orig-alt  3T-orig-viol | -0.27 | -7.55 | <.001** |
| 3T-orig-alt  3T-alt-viol | -0.29 | -7.18 | <.001** |
| 3T-orig-viol  3T-alt-viol | -0.03 | -0.85 | .40 |
| 1T-orig-alt  3T-orig-alt | 0.08 | 2.3 | .03 |
| 1T-orig-viol  3T-orig-viol | 0.04 | 1.29 | .20 |
| 1T-alt-viol  3T-alt-viol | 0.10 | 3.26 | .002* |
| Holm-Bonferroni correction applied for 18 multiple comparisons.  **Significant at <.001, *Significant at <.05. | | | |
